# Genetic deletion of connexin 37 causes polyuria and polydipsia

**DOI:** 10.1371/journal.pone.0244251

**Published:** 2020-12-17

**Authors:** Jianxiang Xue, Linto Thomas, Jessica A. Dominguez Rieg, Robert A. Fenton, Timo Rieg

**Affiliations:** 1 Department of Molecular Pharmacology and Physiology, Morsani College of Medicine, University of South Florida, Tampa, Florida, United States of America; 2 Department of Biomedicine, Aarhus University, Aarhus, Denmark; Anatomy, SWITZERLAND

## Abstract

The connexin 37 (Cx37) channel is clustered at gap junctions between cells in the renal vasculature or the renal tubule where it is abundant in basolateral cell interdigitations and infoldings of epithelial cells in the proximal tubule, thick ascending limb, distal convoluted tubule and collecting duct; however, physiological data regarding its role are limited. In this study, we investigated the role of Cx37 in fluid homeostasis using mice with a global deletion of Cx37 (Cx37^-/-^ mice). Under baseline conditions, Cx37^-/-^ had ~40% higher fluid intake associated with ~40% lower urine osmolality compared to wild-type (WT) mice. No differences were observed between genotypes in urinary adenosine triphosphate or prostaglandin E2, paracrine factors that alter renal water handling. After 18-hours of water deprivation, plasma aldosterone and urine osmolality increased significantly in Cx37^-/-^ and WT mice; however, the latter remained ~375 mmol/kg lower in Cx37^-/-^ mice, an effect associated with a more pronounced body weight loss despite higher urinary AVP/creatinine ratios compared to WT mice. Consistent with this, fluid intake in the first 3 hours after water deprivation was 37% greater in Cx37^-/-^ vs WT mice. Cx37^-/-^ mice showed significantly lower renal AQP2 abundance and AQP2 phosphorylation at serine 256 than WT mice in response to vehicle or dDAVP, suggesting a partial contribution of the kidney to the lower urine osmolality. The abundance and responses of the vasopressin V_2_ receptor, AQP3, NHE3, NKCC2, NCC, H^+^-ATPase, αENaC, γENaC or Na^+^/K^+^-ATPase were not significantly different between genotypes. In summary, these results demonstrate that Cx37 is important for body water handling.

## Introduction

Connexins (Cx) are a family of four-pass transmembrane proteins, which are major gap junction proteins in vertebrates [1]. Six Cx assemble to form a connexon, or hemichannel, and two connexons of adjacent cells interact to form a gap junction channel, which assemble into gap junction plaques. These play important roles in intercellular communication and exchange of small molecules, including ions, metabolites, and second messengers [2,3]. At least 20 connexin isoforms have been identified in humans and rodents [4]. The pathophysiological relevance of Cx are evident from disease causing mutations, with mutations in 10 Cx isoforms underlying 28 genetic diseases [5].

Multiple Cx isoforms, including Cx26, Cx30, Cx30.3, Cx31, Cx32, Cx37, Cx40, Cx43, Cx45, and Cx46 are detected in the kidney [6–15]. Cx37, Cx40, Cx43 and Cx45 are expressed in the renal vasculature, with a species-dependent distribution pattern (for review see [6]). Cx37 is expressed in afferent arterioles; however, functional studies failed to identify a role of Cx37 for renin secretion in response to salt depletion or angiotensin II perfusion [16]. Cx40 is expressed in endothelial cells of arcuate and interlobular arteries, and renin-secreting cells of the afferent arterioles. Functionally, Cx40 is involved in renal autoregulation [17,18], tubuloglomerular feedback (TGF) [19] and renin release [16]. Cx43 is found in various vascular endothelial cells in the kidney [10], where it is hypothesized to modulate renin secretion and blood pressure [20]. Cx45 is expressed in the vascular smooth muscle cells of interlobular arteries, afferent and efferent arterioles and the juxtaglomerular apparatus [18,21,22].

Along the renal tubule epithelium, Cx26, Cx30, Cx30.3, Cx32, Cx37 and Cx43 have been identified. Cx26 and Cx32 are co-expressed in the proximal tubule [23–25], but currently no functional studies have determined their renal role. In rats, Cx30 is expressed from the thick ascending limb of the loop of Henle through the collecting duct, including intercalated cells; however, principal cells were devoid of Cx30 [26]. Cx30 levels are altered by dietary salt [26], and Cx30 plays an integral role in pressure natriuresis by releasing ATP into the tubular fluid, which inhibits salt and water reabsorption [27]. Cx30.3 is expressed in the thin ascending limb of the loop of Henle and in the intercalated cells of the collecting duct [28,29], but its renal role is unknown [29]. Cx32 is observed in mouse proximal tubules [25], and whilst its normal physiological role is not described, knockout of Cx32 attenuated ischemia reperfusion-induced acute kidney injury [30].

Cx37 is one of the most abundantly expressed Cx isoforms throughout the renal vasculature and renal tubule. In the renal tubule, Cx37 is abundant in basolateral cell interdigitations and infoldings of cells in the proximal tubule, thick ascending limb, distal convoluted tubule and collecting duct [31]. A comparison between low and high salt diet showed inverse regulation of Cx37 mRNA and protein expression levels with salt intake [31]. Cx43 is localized to inner medullary collecting ducts of the rat [8] and studies indicate a possible role in the progression of chronic kidney disease [32–34].

Despite its high abundance, the physiological role of Cx37 in the kidney is unclear. In the current study, we describe a novel role of Cx37 for body fluid homeostasis, with Cx37 knockout mice (Cx37^-/-^ mice) having greater fluid intake and lower urine osmolality relative to WT. This phenotype is associated with reduced abundance and phosphorylation of the water channel aquaporin 2 (AQP2).

## Materials and methods

### Animals and ethics

All animal experimentation was conducted in accordance with the Guide for Care and Use of Laboratory Animals (National Institutes of Health, Bethesda, MD) and was approved by the local Institutional Animal Care and Use Committee at the University of South Florida (3338R). The generation of the Cx37^-/-^ mice model has been described previously [35], all animals were bred in-house at the University of South Florida. WT and Cx37^-/-^ mice were housed under a 12:12-hour light-dark cycle in isolated ventilated cages with free access to standard rodent chow (TD.2018; Envigo, Madison, WI) and tap water. A total of 57 age-matched, 3- to 6-month old male and female mice (WT, Cx37^-/-^) were used for experiments.

### Urine and plasma analysis

Fluid and food intake were measured over 9 days in isolated ventilated cages and averaged. Spontaneously voided urine was collected for determination of Na^+^, K^+^, creatinine, urea, and osmolality. Blood was drawn from the retroorbital plexus under brief isoflurane anesthesia (5%) for determination of plasma osmolality and urea. Osmolality was measured by an Osmomat 3000 (Gonotec GmbH, Berlin, Germany), Na^+^ and K^+^ by flame photometry (BWB Technologies Ltd, Berkshire, UK), and creatinine and urea using commercial enzymatic assays (Thermo Fisher Scientific, Waltham, MA). Plasma aldosterone was determined using a solid phase enzyme-linked immunosorbent assay (IBL America, Minneapolis, MN). Urinary arginine-vasopressin and prostaglandin E2 (PGE2) were determined by enzyme-linked immunosorbent assays (Enzo Life Sciences, Farmingdale, NY). Urinary ATP was determined using an ATP colorimetric assay (BioVision, Milpitas, CA).

### Water deprivation

Water deprivation was conducted overnight (18 hours), followed by determination of changes in body weight and collection of spontaneous voided urine to measure osmolality and concentrations of Na^+^, K^+^, urea and creatinine. Blood was taken from the retroorbital plexus for determination of osmolality after brief isoflurane anesthesia. After re-introducing the water bottle, fluid intake was determined in 3-hour intervals for the subsequent 6 hours.

### Response to exogenous activation of vasopressin type 2 receptors (V_2_R)

Mice were treated with 5% dextrose/1% ethanol solution overnight to suppress endogenous arginine-vasopressin (AVP) levels [36,37]. The next day, the mice were intraperitoneally injected with vehicle (sterile water, 2 μl/g bw) or the V_2_R agonist D-amino D-arginine vasopressin (dDAVP) (0.1 μg/kg in sterile water, 2 μl/g bw; Sigma-Aldrich, St. Louis, MO). Mice were observed in their home cages and euthanized by isoflurane overdose (5% until cessation of breathing followed by cervical dislocation as secondary method) 60 minutes after injection, and kidneys were harvested for Western blotting [38].

### Immunoblot analysis

Renal tissue was homogenized in buffer containing protease inhibitor cocktail (Roche Applied Science, Penzberg, Germany) and Halt phosphatase inhibitor cocktail (Thermo Fisher Scientific) as described previously [39,40]. Plasma membrane-enriched samples (by centrifugation at 17,000 x g) were prepared for Western blotting. Proteins were transferred to polyvinylidene difluoride membranes and immunoblotted with AQP2 [36], pS256 AQP2 [41], V_2_R [42], AQP3 [43], NHE3 (AB3085, Millipore, Billerica MA; characterized in [44]), NKCC2 [37], NCC (SPC-402, StressMarq Biosciences, Cadboro Bay, Victoria, BC, Canada; characterized in [45]), H^+^-ATPase [46], αENaC (provided by Johannes Loffing, Institute of Anatomy, Zürich, Switzerland [47]), γENaC [48,49] and Na^+^/K^+^-ATPase (06–520, Merck Millipore, Darmstadt, Germany [48]). Chemiluminescent detection was performed with ECL Plus (Amersham, Piscataway, NJ). Signal intensity in specific bands was quantified using Image Studio Lite (Qiagen, Hilden, Germany) densitometry analysis. Secondary antibodies were from DAKO (Jena, Germany), and sites of antibody/antigen interaction were visualized using the Enhanced Chemiluminescence System (GE Healthcare, Buckinghamshire, Great Britain) and an ImageQuant LAS 4000 imager (GE Healthcare). Coomassie-stained gels were used to adjust for equal protein loading for immunoblotting. Densitometric analyses were performed using Image Studio Lite (Qiagen).

### Statistical analyses

The data are expressed as mean±SEM. Unpaired and paired Student’s *t*-tests were performed, as appropriate, to analyze for statistical differences between groups. All data were analyzed via GraphPad Prism (version 8, San Diego, CA, USA) or SigmaPlot (version 12.5, San Jose, CA, USA). Significance was considered at *P*<0.05.

## Results

### Basal analysis of Cx37^-/-^ mice with free access to fluid and food

**Fig 1:**
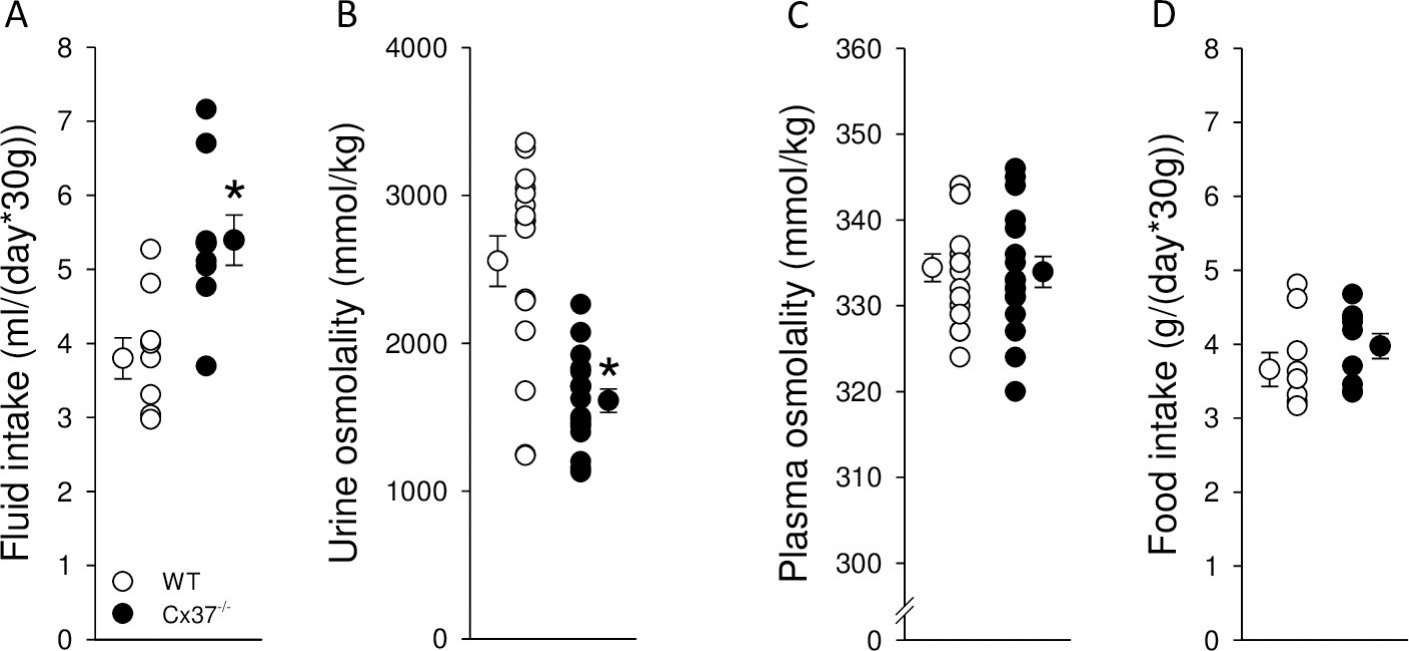
Increased fluid intake and lower urine osmolality in Cx37^-/-^ mice with free access to food and water. (A) Cx37^-/-^ mice have greater fluid intake associated with (B) lower urinary osmolality than WT mice (~142% and ~63% of WT, respectively). Intact water balance is indicated by similar (C) plasma osmolality and (D) food intake between genotypes. Data are expressed as mean ± SEM; *n* = 9 for fluid and food intake, *n* = 12–17 for urine and plasma osmolalities. Data were analyzed by Student’s *t*-test, *P<0.05 *versus* WT.

**Fig 2:**
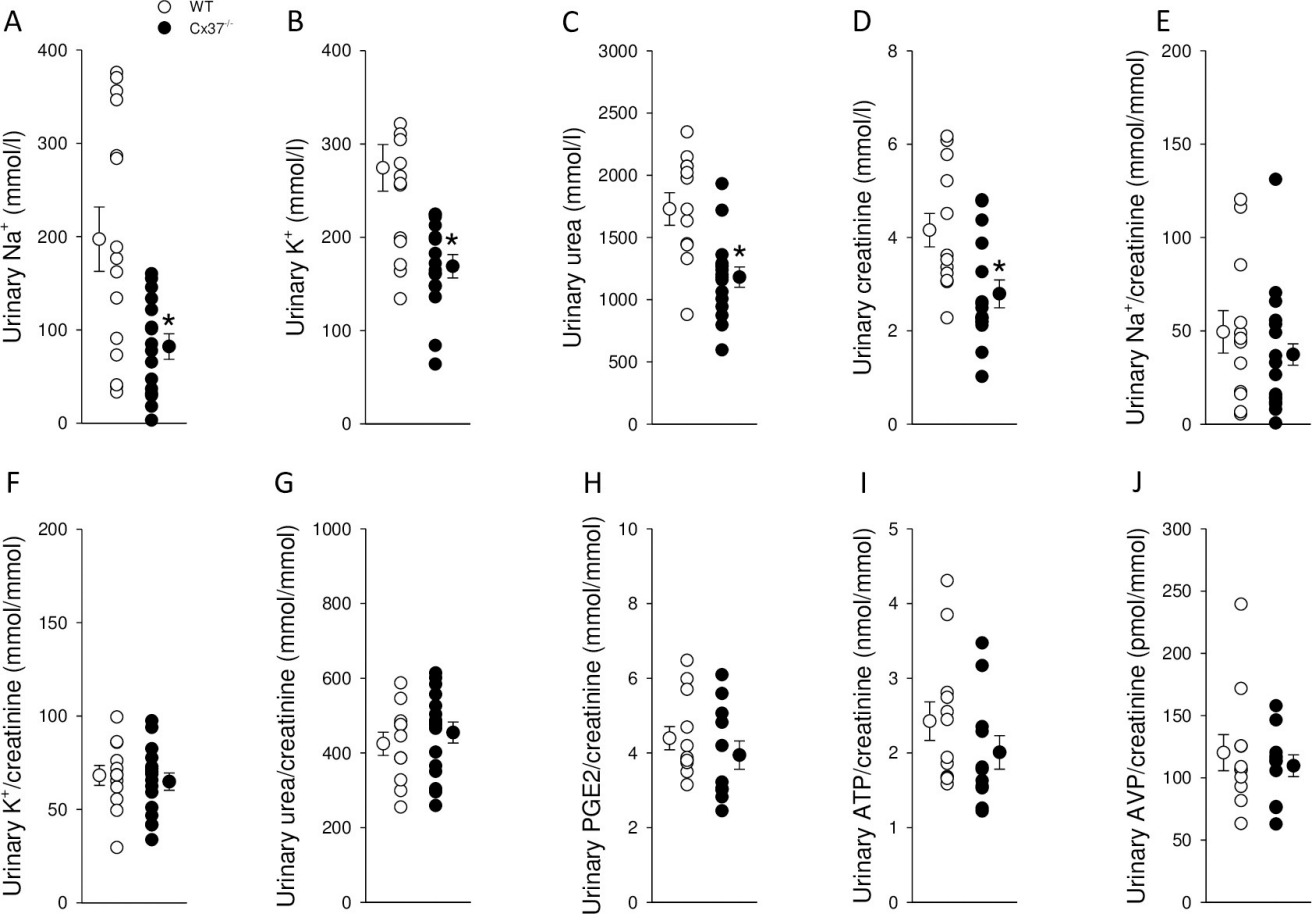
Urinary concentrations of Na^+^, K^+^, urea and creatinine were reduced in Cx37^-/-^ compared to WT mice. Urinary concentrations of (A) Na^+^, (B) K^+^, (C) urea and (D) creatinine were reduced to ~42%, ~62%, ~68% and ~67% in Cx37^-/-^ compared to WT, respectively. Corrected by urinary creatinine no significant differences were observed for (E) Na^+^, (F) K^+^, (G) urea, PGE2 (H), ATP (I) or AVP (J). Data are expressed as mean ± SEM; *n* = 12–17. Data were analyzed by Student’s *t*-test, *P<0.05 *versus* WT.

Under baseline conditions with free access to drinking water, Cx37^-/-^ mice had a ~40% higher fluid intake (Fig 1A) associated with a ~40% lower urine osmolality (Fig 1B) compared to WT mice. Plasma osmolality was not significantly different between genotypes (Fig 1C), indicating that Cx37^-/-^ mice were in fluid balance. No differences in food intake were observed (Fig 1D). Consistent with the lower urine osmolality, Cx37^-/-^ mice had lower urinary concentrations of Na^+^ (Fig 2A), K^+^ (Fig 2B), urea (Fig 2C) and creatinine (Fig 2D). When Na^+^, K^+^ and urea were corrected by urinary creatinine, no significant differences were observed between genotypes (Fig 2E–2G). Urinary PGE2/creatinine (Fig 2H), ATP/creatinine (Fig 2I) and AVP/creatinine ratios (Fig 2J) were not significantly different between genotypes.

The urine-to-plasma ratio for osmolality was reduced by approximately 60% in Cx37^-/-^ compared to WT mice (4.6±0.2 versus 7.4±0.5, *n* = 14–15; *P*<0.05), highlighting polyuria but maintained body water balance. Plasma levels of urea were not different between Cx37^-/-^ and WT mice (10.1±0.3 versus 9.1±0.5 mmol/l, *n* = 14–15; not significant [*NS*]), but urinary urea concentration (Fig 2C) was reduced in the same proportion as urinary osmolality. As observed for the total osmoles, the urine-to-plasma ratio for urea was reduced by approximately 60% in Cx37^-/-^ versus WT mice (119±10 versus 206±24, *n* = 14–15; *P*<0.05).

### Response to water deprivation

**Fig 3:**
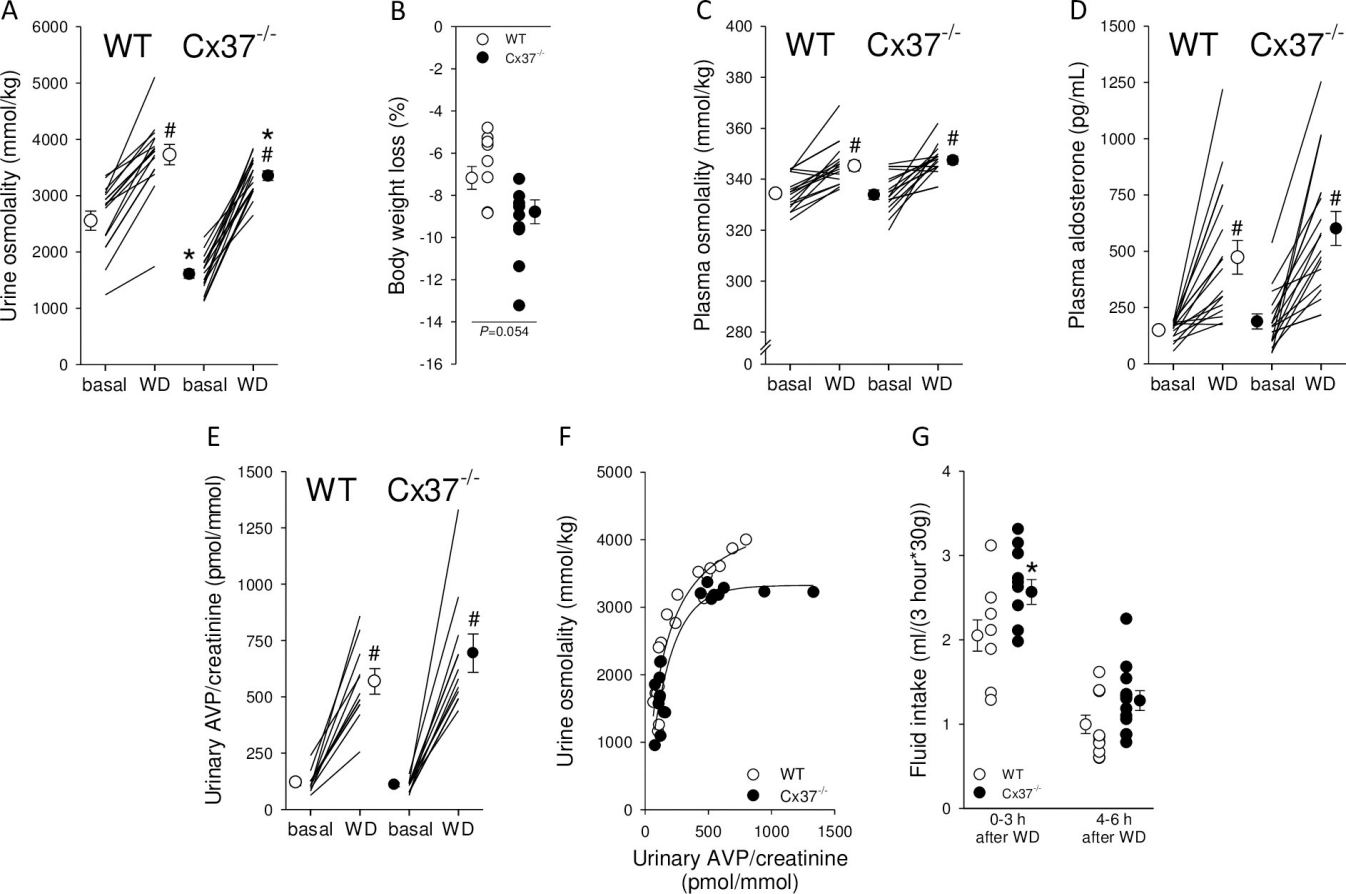
Water homeostasis in WT and Cx37^-/-^ mice in response to 18-hour water deprivation (WD). (A) WD significantly increased urine osmolality in Cx37^-/-^ mice but not to the levels achieved in WT mice, remaining ~10% lower. (B) WD was associated with a tendency for greater loss in body weight. Increases in plasma osmolality (C) and plasma aldosterone (D) were not different between genotypes. (E) There was no difference between genotypes in urinary AVP/creatinine in response to WD compared to baseline. (F) Relationship between urine osmolality and urinary AVP/creatinine ratio between genotypes. Data under baseline conditions and after WD have been plotted. (G) Cx37^-/-^ mice showed ~25% higher fluid intake in the 1^st^ 3 hours after water was re-introduced and a tendency (~28%) for higher fluid intake from 4–6 hours. Data are expressed as mean ± SEM; n = 15–16 for urine osmolality, body weight and plasma osmolality, n = 10–12 for fluid intake and AVP. Data were analyzed by unpaired and paired Student’s *t*-test, *P<0.05 *versus* WT.

**Fig 4:**
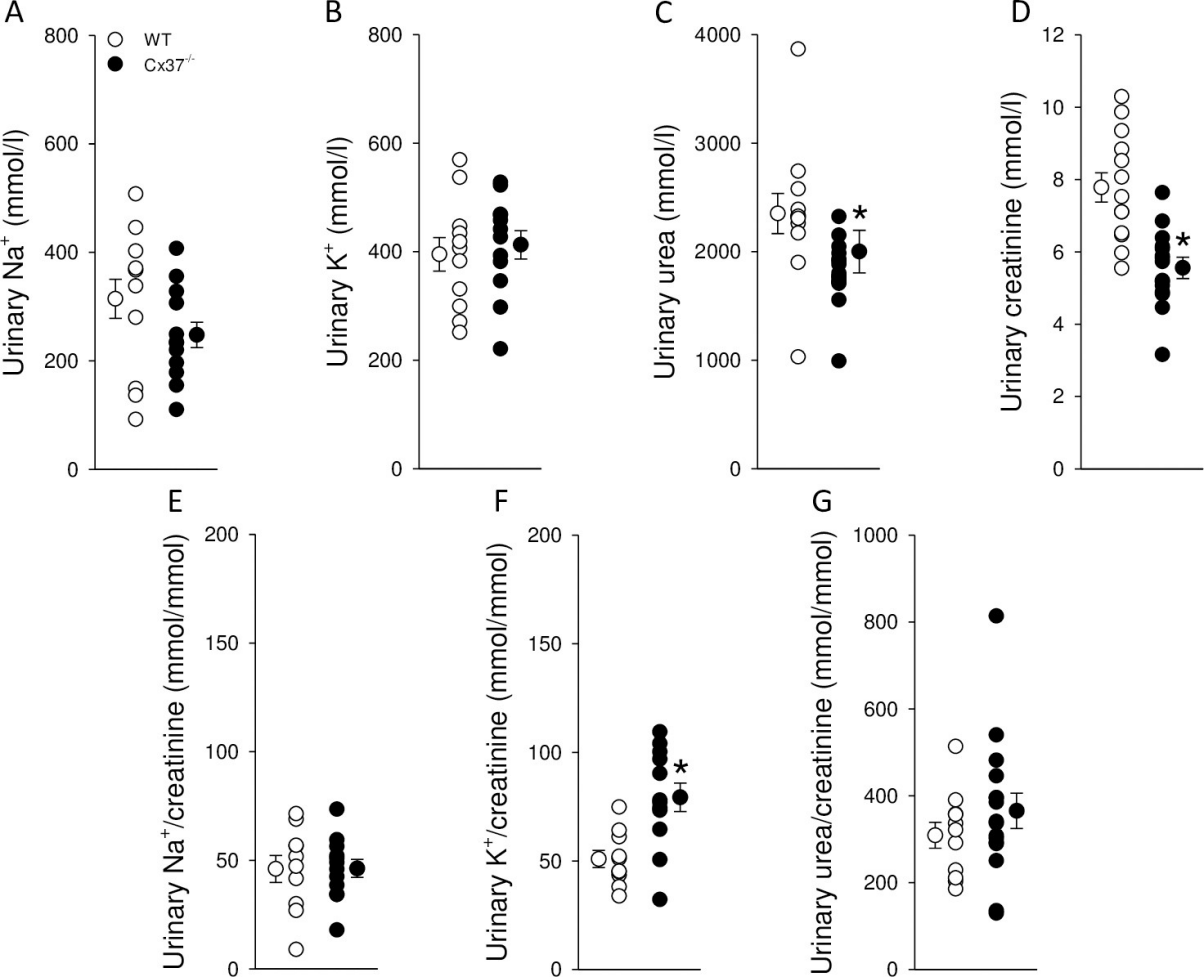
Effects of 18-hour WD on urinary electrolytes and urea in Cx37^-/-^ and WT mice. Urinary concentrations of (A) Na^+^ and (B) K^+^ were not significantly different between genotypes after WD. In contrast, concentrations of (C) urea and (D) creatinine were ~15% and ~29% lower in Cx37^-/-^ compared to WT mice, respectively. Urinary Na^+^/creatinine and urea/creatinine were not different between genotypes (E and G, respectively); however, urinary K^+^/creatinine was significantly higher in in Cx37^-/-^ compared to WT mice (F). Data are expressed as mean ± SEM; n = 11–16. Data were analyzed by Student’s *t*-test, *P<0.05 *versus* WT.

To test for a role of Cx37 in urinary concentrating ability, mice were challenged by an 18-hour water deprivation. Urine osmolality increased significantly in both groups (Fig 3A) but remained ~375 mmol/kg lower in Cx37^-/-^ compared to WT mice. This was associated with a tendency for greater body weight loss (Fig 3B). In response to water deprivation, both genotypes showed a significant increase in plasma osmolality (Fig 3C) but the increase was not significantly different between Cx37^-/-^ and WT mice (Δ change: 14±3 versus 11±2 mmol/kg, *NS*). Baseline plasma aldosterone levels and alterations in response to water deprivation (Fig 3D) were comparable between genotypes (Δ change: 413±69 versus 326±71 pg/mL, *NS*). The increase in urinary AVP/creatinine in response to water deprivation compared to baseline (Fig 3E) was not significantly different between WT and Cx37^-/-^ mice (5±1 and 6±1 fold, *NS*). Despite Cx37^-/-^ mice reaching a maximum urine osmolality of ~3250 mmol/kg they required higher urinary AVP/creatinine levels (Fig 3F). Fluid intake, up to 3 hours after water deprivation, was ~25% greater in Cx37^-/-^ compared to WT mice (Fig 3G). This difference decreased in the next 3 hours. Urinary concentrations of Na^+^ and K^+^ were not significantly different between genotypes after water deprivation (Fig 4A and 4B); however, urinary urea (Fig 4C) and creatinine (Fig 4D) concentrations remained significantly lower in Cx37^-/-^ compared to WT mice. When corrected by urinary creatinine, the ratios for Na^+^ (Fig 4E) and urea (Fig 4G) were not significantly different between genotypes, whereas urinary K^+^/creatinine was significantly higher in Cx37^-/-^ compared to WT mice (Fig 4F).

### Effect of DAVP on AQP2 abundance and phosphorylation in Cx37^-/-^ mice

**Fig 5:**
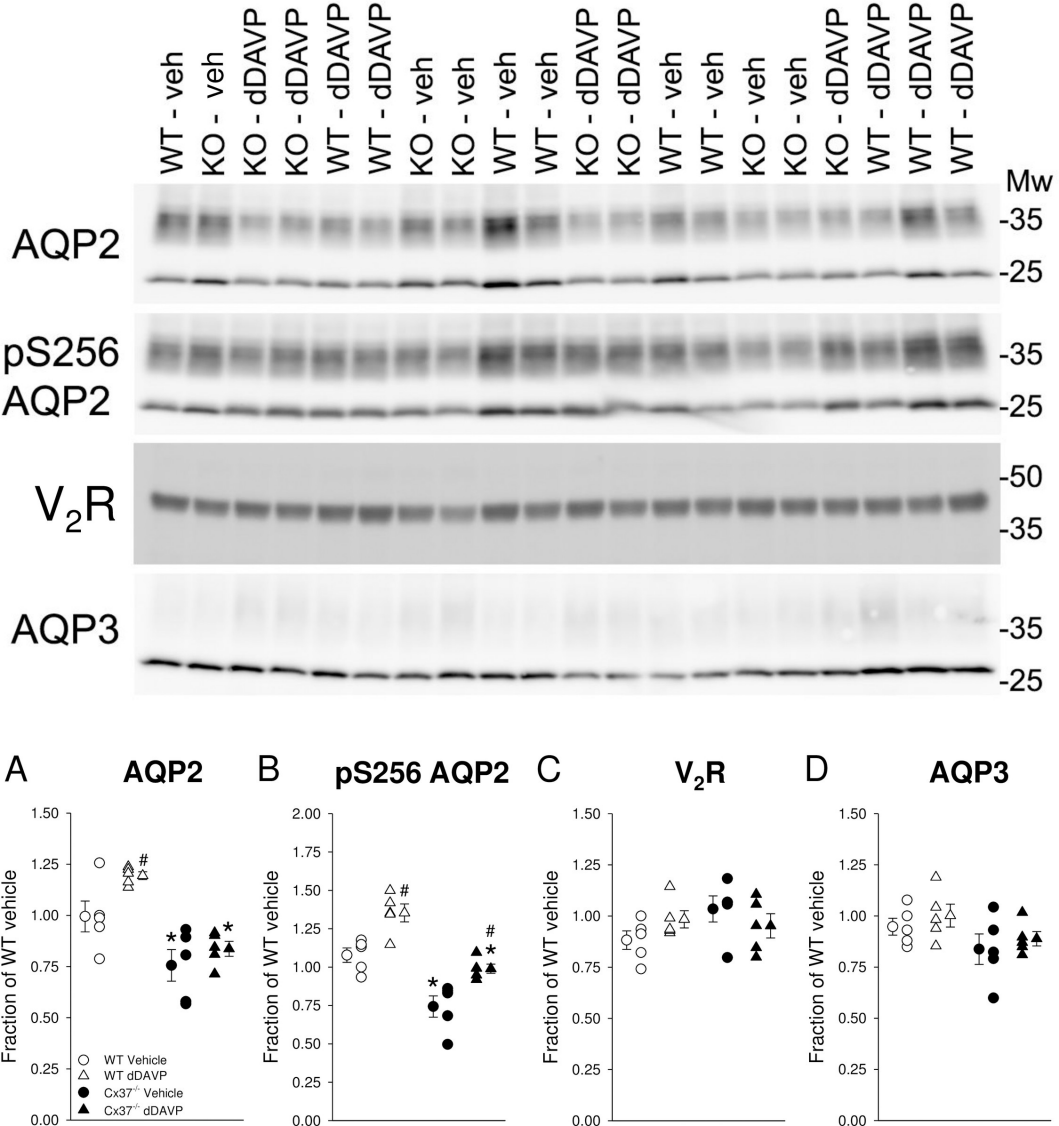
Renal abundance and phosphorylation of AQP2 is reduced under vehicle and impaired in Cx37^-/-^ compared to WT mice in response to V_2_R activation. Sixty minutes after vehicle application Cx37^-/-^ mice had lower abundance of (A) total and (B) phosphorylated serine 256 AQP2 compared with WT mice. Systemic administration of dDAVP following suppression of endogenous arginine-vasopressin overnight (see Materials and methods for details) increased the abundance of (A) total and (B) phosphorylated serine 256 AQP2 in both genotypes; however, the effect was attenuated in Cx37^-/-^ mice. No differences in the abundance of V_2_R or AQP3 were observed between genotypes or treatments. Data are expressed as mean ± SEM; *n* = 5 each genotype and condition. Data were analyzed by paired and unpaired Student’s *t*-test, *P<0.05 *versus* WT same condition, ^#^*P*<0.05 *versus* vehicle same genotype.

To study the *in vivo* response to V_2_R activation, we measured the abundance and phosphorylation of AQP2 by Western blotting in response to dDAVP or vehicle treatment after suppression of endogenous AVP by ethanol/water loading overnight. This maneuver caused a significant reduction of urine osmolality that was not different between genotypes (WT: 232±43 versus Cx37^-/-^: 189±24 mmol/kg, *NS*). Due to the short duration of the dDAVP stimulation, 60 minutes, we were not able to collect enough urine for analysis. With vehicle, in kidney lysates enriched for plasma membranes, total AQP2 expression was significantly lower in Cx37^-/-^ versus WT mice (Fig 5A). WT mice responded to dDAVP with an increase in total AQP2 abundance. This response was limited in Cx37^-/-^ mice (Fig 5A). Consistent with total AQP2, pS256-AQP2 abundance was significantly lower in Cx37^-/-^ compared to WT mice in response to vehicle application (Fig 5B). Both genotypes showed a significant increase in pS256-AQP2 abundance (an important site for AQP2 plasma membrane targeting in response to dDAVP [50]; however, the effect in Cx37^-/-^ mice remained significantly lower compared to WT mice (Fig 5B). These differences were not caused by differences or changes in V_2_R abundances (Fig 5C). AQP3 abundance (expressed on the basolateral membrane of principal cells) was not significantly different between genotypes in response to vehicle application and no changes in AQP3 abundance in response to dDAVP were observed (Fig 5D).

### Responses of renal salt transporters and channels in WT and Cx37^-/-^ mice to dDAVP

**Fig 6:**
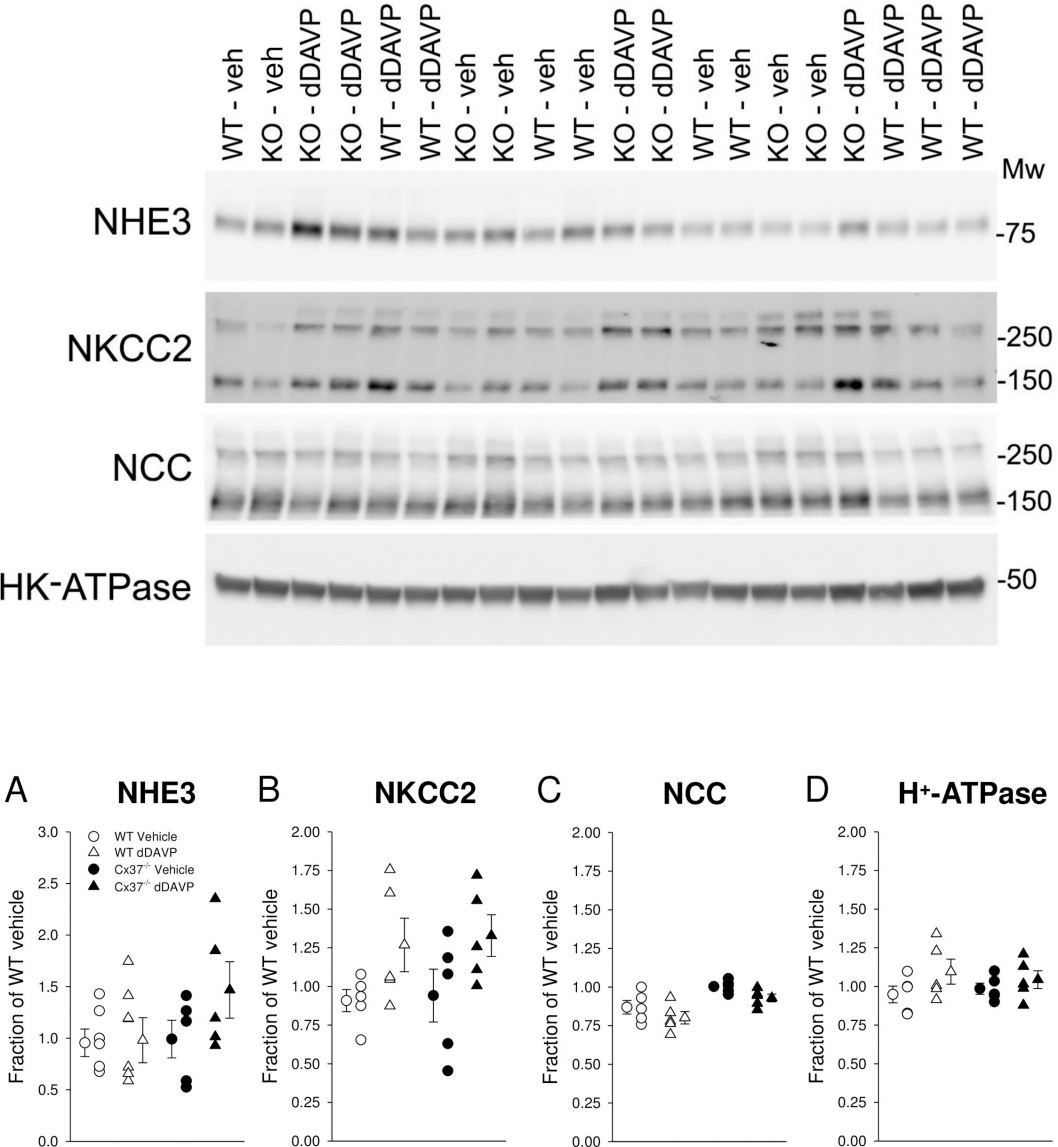
Renal abundance of major salt and proton transporting proteins. Sixty minutes after vehicle application no differences were observed in (A) NHE3, (B) NKCC2, (C) NCC or (D) H^+^-ATPase abundance. Systemic administration of dDAVP following suppression of endogenous vasopressin overnight (see Materials and methods for details) did not affect abundances of these proteins. Data are expressed as mean ± SEM and analyzed by paired and unpaired Student’s *t*-test, *n* = 5 each genotype and condition.

**Fig 7:**
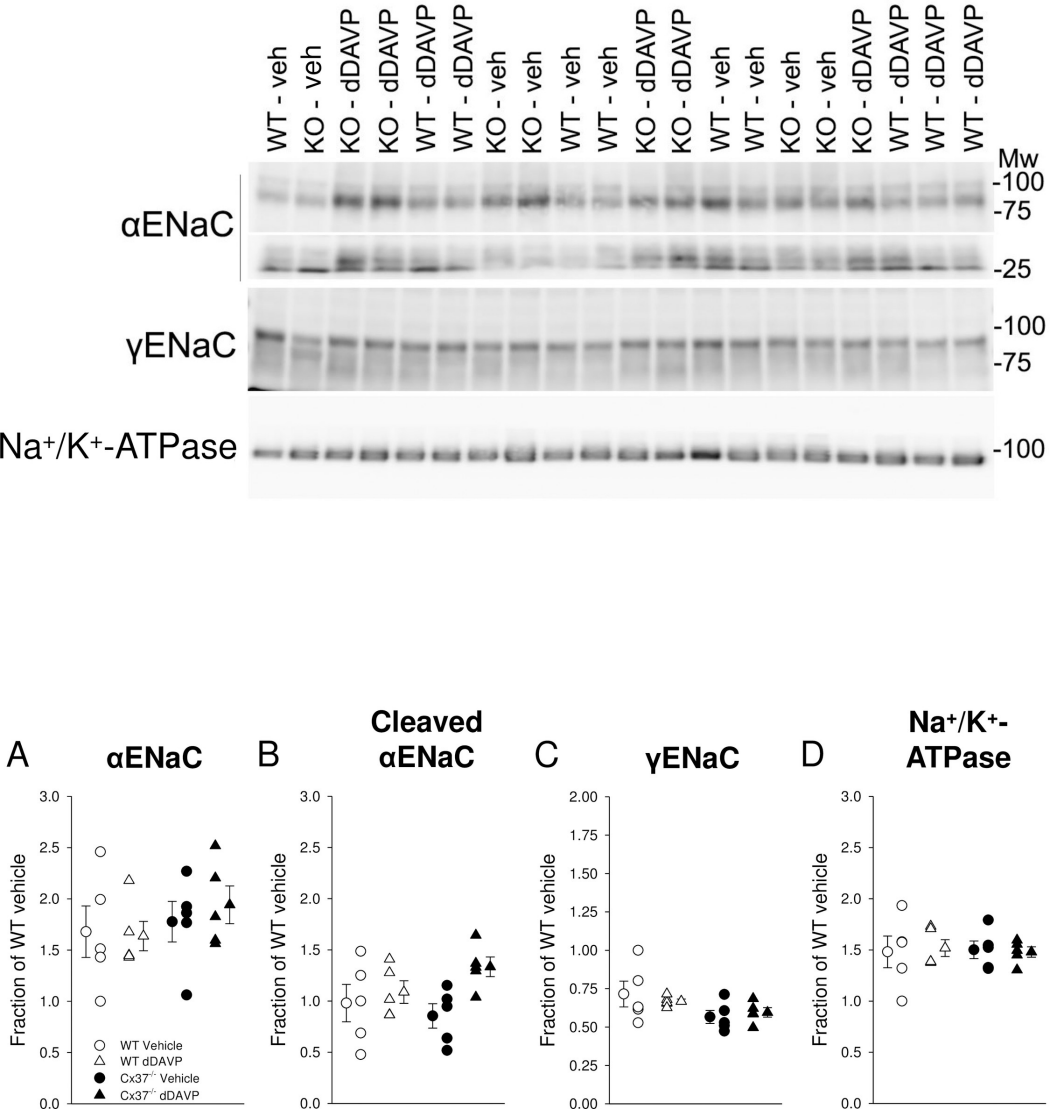
Renal abundance of the epithelial sodium channel and Na^+^/K^+^-ATPase. Sixty minutes after vehicle application no differences were observed in (A) αENaC, (B) cleaved αENaC, (C) γENaC or (D) Na^+^/K^+^-ATPase abundance. Systemic administration of dDAVP following suppression of endogenous arginine-vasopressin overnight (see Materials and methods for details) did not affect abundances of these proteins. Data are expressed as mean ± SEM and analyzed by paired and unpaired Student’s *t*-test, *n* = 5 each genotype and condition.

In order to determine if reduced abundance of other transporters and channels that may be involved in renal salt and water handling could explain the water handling defect, the major salt transporters and channels along the nephron were profiled in kidney lysates enriched for plasma membranes. NHE3 abundance, mainly expressed in the proximal tubule and thick ascending limb (TAL) [51], was not significantly different between genotypes after vehicle application and no dDAVP effects were observed (Fig 6A). NKCC2 abundance, mainly expressed in the TAL, was not significantly different after vehicle application (Fig 6B). dDAVP was previously shown to regulate NKCC2 in the renal medulla [37] and both genotypes showed tendencies for increased NKCC2 abundance that did not reach statistical significance (Fig 6B). NCC, expressed in the distal convoluted tubule, was not significantly different between genotypes after vehicle application and no dDAVP effects were observed (Fig 6C). Similar findings were observed for H^+^-ATPase (Fig 6D; expressed in the proximal tubule and collecting duct), α-subunit of ENaC (Fig 7A; principal cells), cleaved α-subunit of ENaC (Fig 7B; indicative of proteolytic activation), γ-subunit of ENaC (Fig 7C; principal cells), and Na^+^/K^+^-ATPase (Fig 7D; expressed in the basolateral membrane).

## Discussion

Connexins play critical roles in intercellular communication, e.g. electrical coupling, autocrine/paracrine signaling, and development. Cx37 is expressed in the kidney; however, little is known about its physiological function(s). Classical gap junctions have only been localized (using freeze fracture) in the proximal tubule of rat [52] and human kidneys [53], implying that Cx37 along the further distal segments and collecting ducts rather function as hemichannels [6]. The major finding in this study is that we show for the first time that Cx37 contributes to water homeostasis *in vivo*. Cx37^-/-^ mice have higher fluid intake and lower urine osmolality, but Cx37^-/-^ mice are in fluid balance, with no signs of plasma electrolyte defects or growth retardation. So what is the basis for the alteration in fluid intake and urine volume–is it of renal or of central origin [54]? One way to differentiate between the two possibilities is based on plasma osmolality, which in patients with nephrogenic diabetes insipidus (NDI) is either normal [36,41,55] or slightly increased [56–58], and in primary polydipsia is often lower [59]. However, mice with primary polydipsia do not show differences in plasma osmolality [60,61]. Based on our studies, we hypothesize that the phenotype in Cx37^-/-^ mice is more consistent with primary polydipsia.

To categorically determine if Cx37^-/-^ mice had NDI, we assessed the response of these mice to water deprivation. After water deprivation, although the urine osmolality of Cx37^-/-^ mice was still significantly lower than WT mice, accompanied by a tendency for greater body weight loss, the Cx37^-/-^ mice still concentrated their urine to > 3000 mmol/kg suggesting an intact urinary concentrating mechanism. However, the Cx37^-/-^ mice do not concentrate their urine above ~3250 mmol/kg despite higher AVP levels. The increased fluid intake for 3 hours proceeding water deprivation fits with these mice having either to initially compensate more than WT mice to the water deprivation (as their maximal urine concentration is less), or that the reintroduction of water immediately stimulates their compulsive water drinking–or it could be a combination of both. Of note, although the phenotype of Cx37^-/-^ mice fits better with primary polydipsia, Cx37 has not been identified in cells with neuronal morphology in the brain [62]. The Cx37^-/-^ phenotype is also not likely related to increased salt appetite [63] because urinary Na^+^/creatinine ratios and expression of αENaC and proteolytically cleaved αENaC were not significantly different between genotypes. Along those lines, we did not observe differences in plasma aldosterone levels before and in response to water deprivation between genotypes, excluding that aldosterone accounts for differences in urinary K^+^/creatinine in response to water deprivation. If changes in Cx37-mediated Ca^2+^-signaling affects K^+^ secretion via Ca^2+^-dependent big-conductance potassium channels [64], a channel activated by increases in flow, remains to be determined.

Changes in collecting duct water permeability, mediated by AVP-induced AQP2 trafficking, play a critical role in urinary concentration and water balance [65–67]. This process involves adenylyl cyclase 6 (AC6)-mediated cAMP formation in principal cells [36,41], alongside other mechanisms [68] such as enhanced AQP2 phosphorylation [50]. The importance of serine 256 AQP2 phosphorylation in membrane targeting is highlighted in mice with a substitution of serine with a leucine at residue 256, which lack any form of AQP2 phosphorylation and have NDI [69]. The reduced total AQP2 and phosphorylated pS256-AQP2 abundance in Cx37^-/-^ mice fits with the lower baseline urine osmolality, but whether this is the cause–or an effect–is complicated by AQP2 being stimulated by interstitial osmolality [70] that is predicted to be lower in Cx37^-/-^ mice. Of note, the phenotype is consistent with other studies reporting that AQP2 expression is reduced in primary polydipsia [71,72] without affecting V_2_R expression [72]. PGE2 is an important regulator of urinary concentrating ability [73,74] and Cx43 has been shown to regulate PGE2 release from osteocytes [75]. Our data do not show differences in urinary PGE2/creatinine ratios between genotypes, suggesting that PGE2 in Cx37^-/-^ mice does not contribute to the differences in fluid homeostasis. In line with this, differences in V_2_R expression can be excluded to contribute to these differences because no differences in the expression levels were observed between genotypes. Eighteen-hour water restriction resulted in urine osmolarities that were slightly but significantly lower in Cx37^-/-^ versus WT mice, which could be explained by lower total AQP2 and lower phosphorylation of S256-AQP2 in response to dDAVP. A clear response to dDAVP indicates again that the mice do not have NDI. Of note, treating mice with 5% dextrose/1% ethanol solution should completely suppress endogenous vasopressin levels; this maneuver was not intended to dissect between NDI versus primary polydipsia but rather to eliminate differences in endogenous AVP levels between genotypes. The data from our experiments show similarities to other models with defects in water handling but with partially preserved urinary concentrating ability, e.g. AC6 knockout mice [36,41], glycogen synthase kinase 3β knockout mice [76] or calcineurin Aα knockout mice [57].

If not solely compulsive water drinking, what else may contribute to the higher fluid intake and lower urine osmolality in Cx37^-/-^ mice? Connexins and pannexins are also involved in the release of adenosine triphosphate (ATP) and uridine triphosphate (UTP) [6,77], e.g. ATP release through Cx37 occurs in monocytes [78]; however, our data do not show differences in urinary ATP/creatinine ratios. We have previously shown that ATP can inhibit vasopressin-induced cAMP formation and urinary concentrating ability; a process mediated by P2Y_2_ receptors [79]. However, our current data argue against a primary role of Cx37-mediated ATP/UTP release because Cx37^-/-^ mice show *lower* and not *higher* urine osmolality. Likewise, the relatively normal abundance of major transporters and channels involved in renal salt and water handling excludes a major role of these in the observed phenotype. If differences in crosstalk between principal cell and intercalated cells exist in Cx37^-/-^ mice remains to be determined. Our data show that urinary ATP/creatinine ratios were comparable between genotypes; however, Cx30-mediated ATP release from intercalated cells has been shown to affect ENaC open probability in neighboring principal cells [27].

Another major contributor to body water balance is the renal handling of urea [80]. However, as urinary urea concentrations increased in response to water deprivation in both genotypes and there are no differences in urinary urea to total osmolality ratios, altered urea handling is unlikely to be involved. Cx37 polymorphisms can affect endothelial cell function [81,82]. Based on the expression of Cx37 in the vasculature [82], in particular the descending vasa recta [31], we cannot exclude that changes in local renal blood flow also alter body water handling and further studies are needed to better understand the contribution of Cx37 in the vasculature. Of note, no differences in renal blood flow were found between Cx37^-/-^ and WT mice [83].

In summary, this study shows for the first time that Cx37 plays a role in body water handling, with deletion of Cx37 resulting in polyuria and polydipsia. Some of these effects may be mediated by lower AQP2 abundance and phosphorylation at serine 256.

## Supporting information

S1 File (PPTX)
