# Supplementary material for: Genetic deletion of connexin 37 causes polyuria and polydipsia
Source: PLoS One. 2020 Dec 17;15(12):e0244251. doi: 10.1371/journal.pone.0244251 (PMC7746157; doi:10.1371/journal.pone.0244251)
Supplement: S1 File — (PPTX) [file pone.0244251.s001.pptx]

## Slide 1
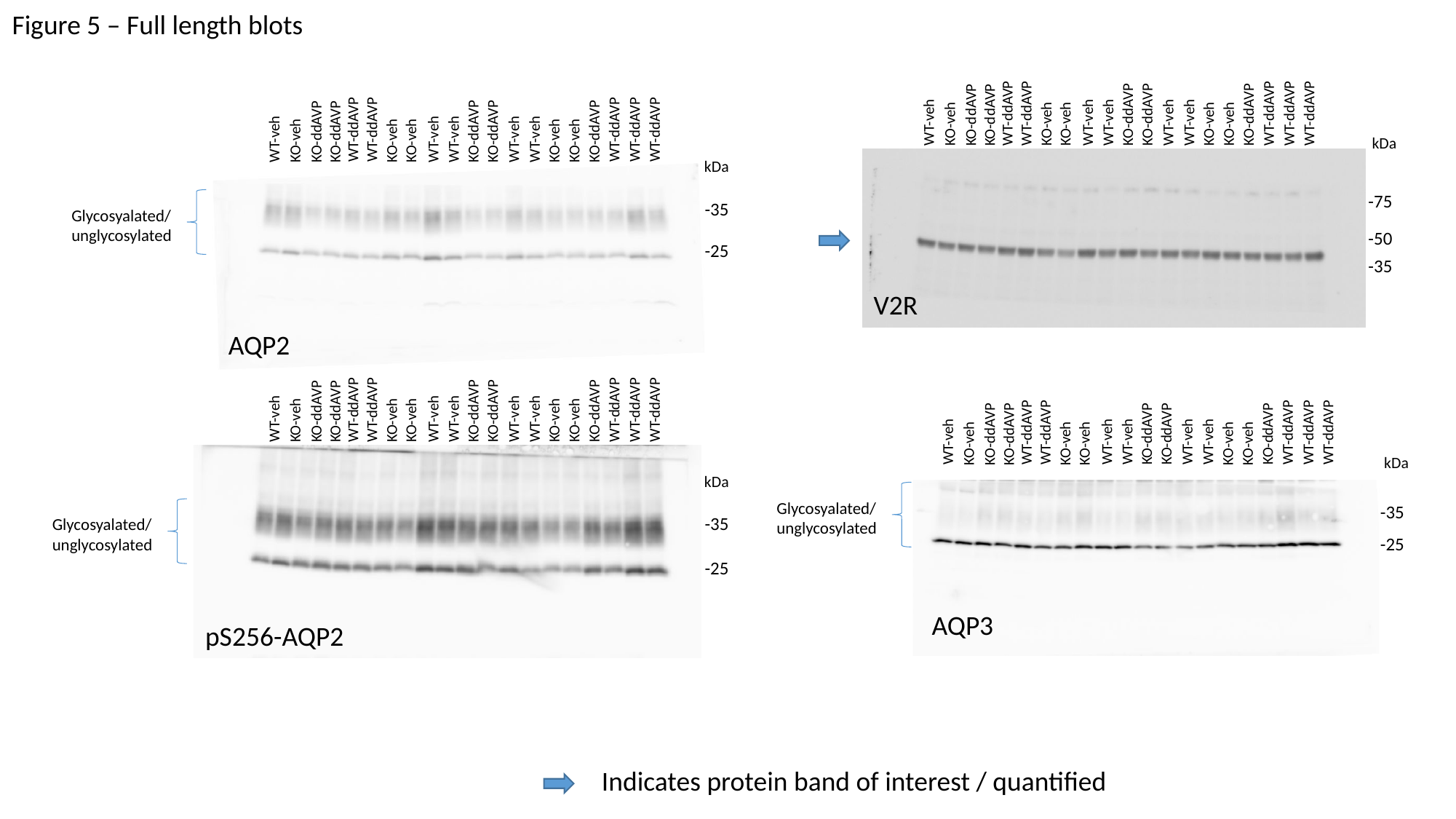

Figure 5 – Full length blots
WT-ddAVP
WT-ddAVP
WT-ddAVP
WT-ddAVP
WT-ddAVP
KO-ddAVP
KO-ddAVP
KO-ddAVP
KO-ddAVP
KO-ddAVP
WT-veh
WT-veh
WT-veh
WT-veh
WT-veh
KO-veh
KO-veh
KO-veh
KO-veh
KO-veh
WT-ddAVP
WT-ddAVP
WT-ddAVP
WT-ddAVP
WT-ddAVP
KO-ddAVP
KO-ddAVP
KO-ddAVP
KO-ddAVP
KO-ddAVP
WT-veh
WT-veh
WT-veh
WT-veh
WT-veh
KO-veh
KO-veh
KO-veh
KO-veh
KO-veh
kDa
kDa
-75
-35
Glycosyalated/
unglycosylated
-50
-25
-35
V2R
AQP2
WT-ddAVP
WT-ddAVP
WT-ddAVP
WT-ddAVP
WT-ddAVP
KO-ddAVP
KO-ddAVP
KO-ddAVP
KO-ddAVP
KO-ddAVP
WT-veh
WT-veh
WT-veh
WT-veh
WT-veh
KO-veh
KO-veh
KO-veh
KO-veh
KO-veh
WT-ddAVP
WT-ddAVP
WT-ddAVP
WT-ddAVP
WT-ddAVP
KO-ddAVP
KO-ddAVP
KO-ddAVP
KO-ddAVP
KO-ddAVP
WT-veh
WT-veh
WT-veh
WT-veh
WT-veh
KO-veh
KO-veh
KO-veh
KO-veh
KO-veh
kDa
kDa
Glycosyalated/
unglycosylated
-35
-35
Glycosyalated/
unglycosylated
-25
-25
AQP3
AQP3
pS256-AQP2
Indicates protein band of interest / quantified

## Slide 2
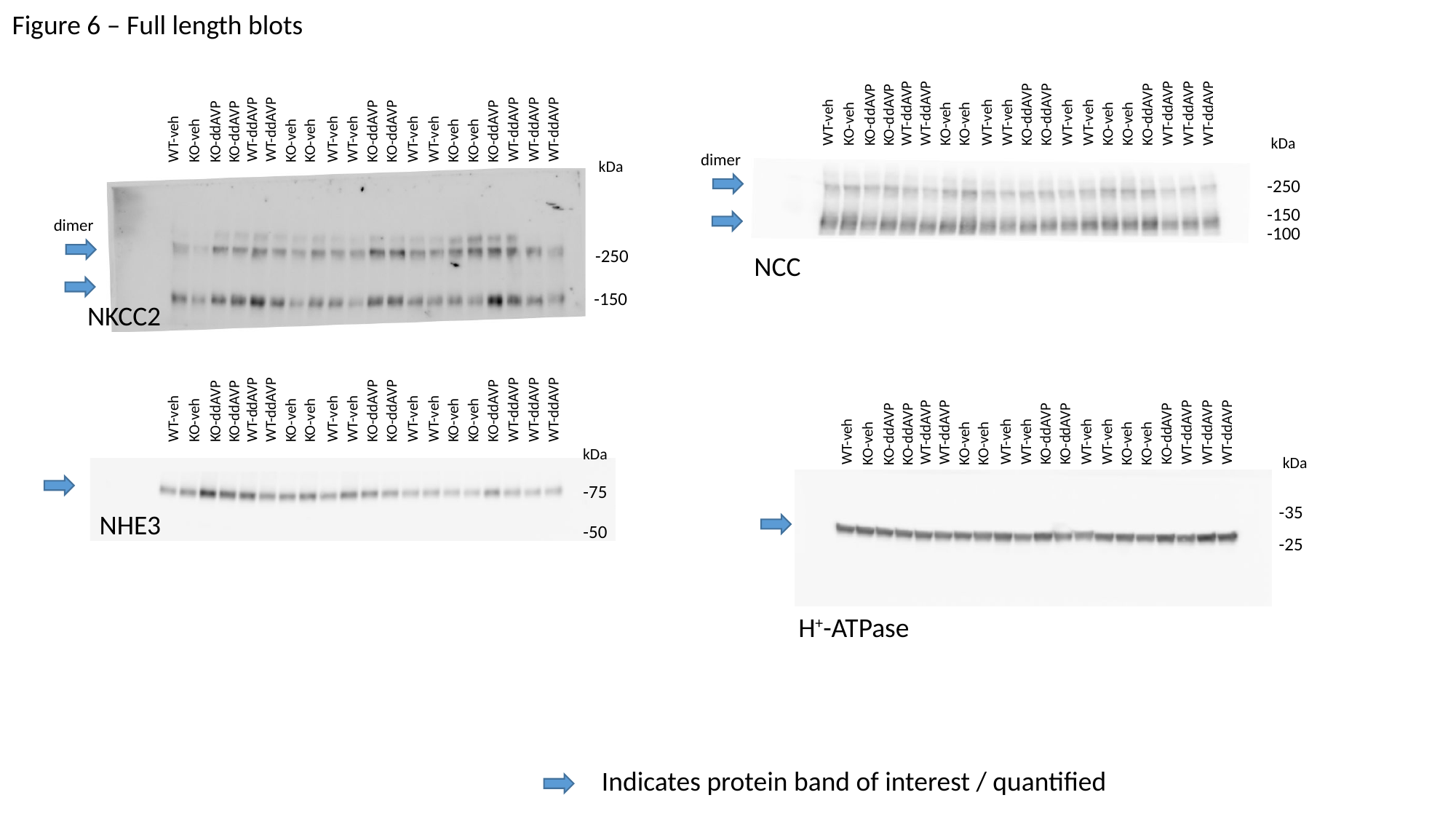

Figure 6 – Full length blots
WT-ddAVP
WT-ddAVP
WT-ddAVP
WT-ddAVP
WT-ddAVP
KO-ddAVP
KO-ddAVP
KO-ddAVP
KO-ddAVP
KO-ddAVP
WT-veh
WT-veh
WT-veh
WT-veh
WT-veh
KO-veh
KO-veh
KO-veh
KO-veh
KO-veh
WT-ddAVP
WT-ddAVP
WT-ddAVP
WT-ddAVP
WT-ddAVP
KO-ddAVP
KO-ddAVP
KO-ddAVP
KO-ddAVP
KO-ddAVP
WT-veh
WT-veh
WT-veh
WT-veh
WT-veh
KO-veh
KO-veh
KO-veh
KO-veh
KO-veh
kDa
dimer
kDa
-250
-150
dimer
-100
-250
NCC
-150
NKCC2
WT-ddAVP
WT-ddAVP
WT-ddAVP
WT-ddAVP
WT-ddAVP
KO-ddAVP
KO-ddAVP
KO-ddAVP
KO-ddAVP
KO-ddAVP
WT-veh
WT-veh
WT-veh
WT-veh
WT-veh
KO-veh
KO-veh
KO-veh
KO-veh
KO-veh
WT-ddAVP
WT-ddAVP
WT-ddAVP
WT-ddAVP
WT-ddAVP
KO-ddAVP
KO-ddAVP
KO-ddAVP
KO-ddAVP
KO-ddAVP
WT-veh
WT-veh
WT-veh
WT-veh
WT-veh
KO-veh
KO-veh
KO-veh
KO-veh
KO-veh
kDa
kDa
-75
-35
NHE3
-50
-25
H+-ATPase
Indicates protein band of interest / quantified

## Slide 3
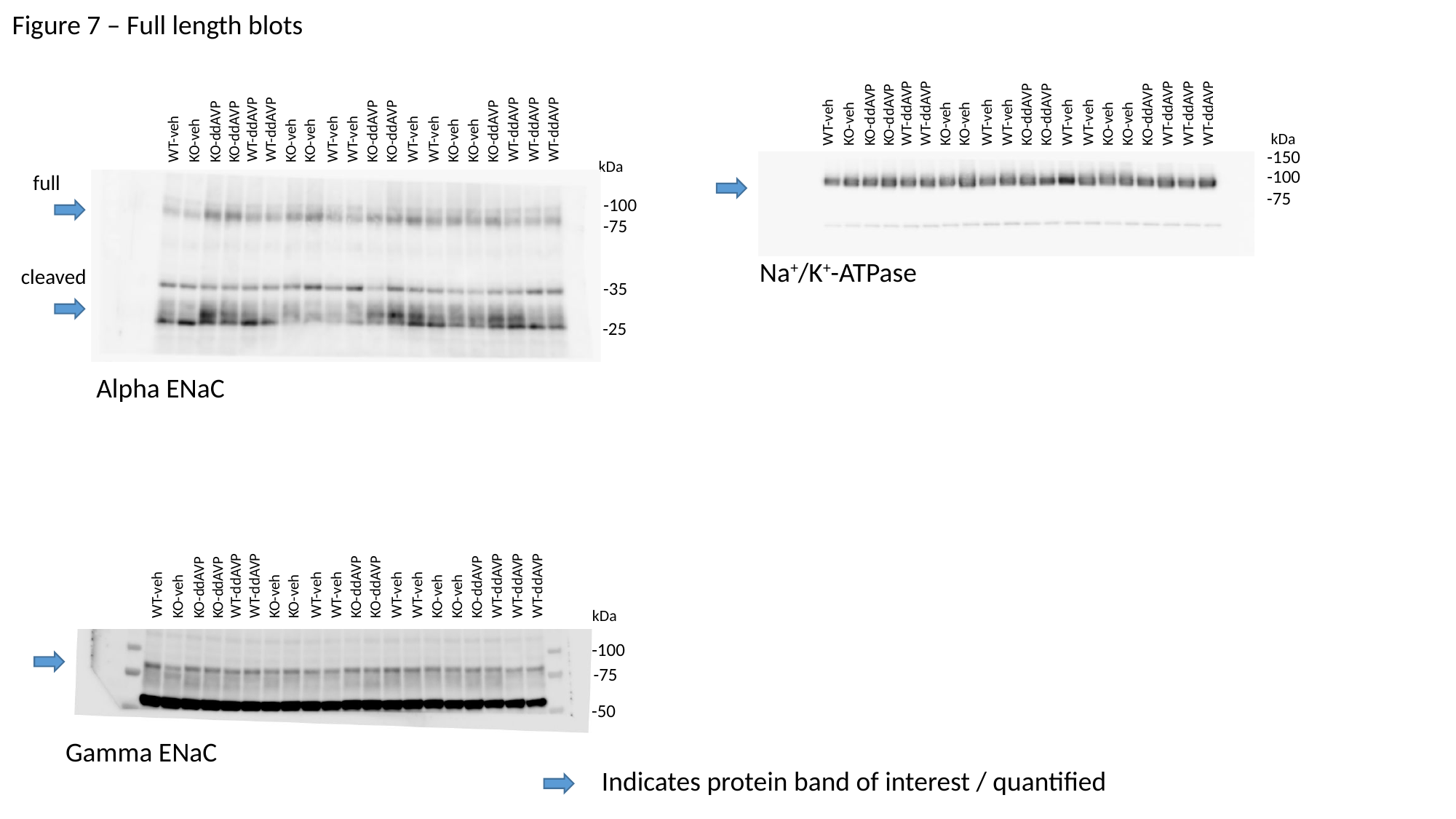

Figure 7 – Full length blots
WT-ddAVP
WT-ddAVP
WT-ddAVP
WT-ddAVP
WT-ddAVP
KO-ddAVP
KO-ddAVP
KO-ddAVP
KO-ddAVP
KO-ddAVP
WT-veh
WT-veh
WT-veh
WT-veh
WT-veh
KO-veh
KO-veh
KO-veh
KO-veh
KO-veh
WT-ddAVP
WT-ddAVP
WT-ddAVP
WT-ddAVP
WT-ddAVP
KO-ddAVP
KO-ddAVP
KO-ddAVP
KO-ddAVP
KO-ddAVP
kDa
WT-veh
WT-veh
WT-veh
WT-veh
WT-veh
KO-veh
KO-veh
KO-veh
KO-veh
KO-veh
-150
kDa
-100
full
-75
-100
-75
Na+/K+-ATPase
cleaved
-35
-25
Alpha ENaC
WT-ddAVP
WT-ddAVP
WT-ddAVP
WT-ddAVP
WT-ddAVP
KO-ddAVP
KO-ddAVP
KO-ddAVP
KO-ddAVP
KO-ddAVP
WT-veh
WT-veh
WT-veh
WT-veh
WT-veh
KO-veh
KO-veh
KO-veh
KO-veh
KO-veh
kDa
-100
-75
-50
Gamma ENaC
Indicates protein band of interest / quantified
